# Association of Peptic Ulcer Disease and Gastrointestinal Hemorrhage With Long-Term Use of Antiplatelet and Anticoagulant Therapy in Patients With Ischemic Heart Disease

**DOI:** 10.7759/cureus.100702

**Published:** 2026-01-03

**Authors:** Kanav Jain, Maria Shaikh, Ayesha Shahid Butt, Mian Mubeen Mansha, Nikhil Charles Madathiparambu, Aliha Rizwan, Shoaib Younas, Muhammad Ali Zaib Khan

**Affiliations:** 1 Internal Medicine, Countess of Chester Hospital NHS Foundation Trust, Chester, GBR; 2 Internal Medicine, South Tipperary General Hospital, Clonmel, IRL; 3 Medical Education, Rashid Latif Medical College, Lahore, PAK; 4 General Practice, Shahina Jamil Teaching Hospital, Abbottabad, PAK; 5 Gastroenterology, Queen Elizabeth Hospital, Broadstairs, GBR; 6 Anesthesia and Critical Care, Amna Inayat Medical College, Sheikhupura, PAK; 7 General Practice, Basic Health Unit (BHU) Bindian, Azad Kashmir, PAK; 8 General Practice, Baba Bulleh Shah (District Headquarters, DHQ) Hospital, Kasur, PAK

**Keywords:** anticoagulant therapy, antiplatelet therapy, ischemic heart disease, peptic ulcer disease, risk factors

## Abstract

Background and aim

Antiplatelet and anticoagulant therapies are essential in the long-term management of ischemic heart disease (IHD). However, their use is associated with increased gastrointestinal (GI) complications, particularly peptic ulcer disease (PUD) and hemorrhage. The aim of the study was to determine the association of peptic ulcer disease and gastrointestinal hemorrhage with long-term use of antiplatelet and anticoagulant therapy in patients with ischemic heart disease.

Methodology

This was a cross-sectional analytical study conducted at a tertiary care hospital in Azad Kashmir, Pakistan, from June 2024 to May 2025. A total of 210 patients receiving antiplatelet and/or anticoagulant therapy for at least six months were enrolled. Data on demographics, comorbidities, therapy category (single, dual, or combined antiplatelet-anticoagulant therapy), drug types, dosages, adherence, nonsteroidal anti-inflammatory drug (NSAID) exposure, H. pylori status, smoking, alcohol intake, proton pump inhibitors (PPI) use, and renal/hepatic disease were collected to assess potential confounders. Only patients presenting with upper GI symptoms or suspected bleeding were referred for endoscopy; thus, asymptomatic PUD may be underestimated. Endoscopic evaluations followed standardized criteria: PUD was diagnosed based on mucosal ulceration ≥5 mm, and hemorrhage severity was classified using the Forrest criteria. Missing data were minimized through direct patient interviews; when unavoidable, listwise deletion was applied. No sensitivity analyses were performed.

Results

Out of 210 patients, 64 (30.5%) were diagnosed with peptic ulcer disease, and 49 (23.3%) developed GI hemorrhage. The highest bleeding frequency was observed in patients receiving combined antiplatelet and anticoagulant therapy (45%, n=18), compared with dual antiplatelet therapy (21.9%, n=23) and single antiplatelet therapy (12.3%, n=8; p<0.01). Logistic regression identified prior ulcer history (OR: 2.6, 95% CI: 1.3-5.1, p=0.004), NSAID use (OR: 3.1, 95% CI: 1.5-6.4, p=0.002), and combined therapy (OR: 4.2, 95% CI: 2.1-8.4, p<0.001) as independent predictors of GI hemorrhage.

Conclusion

Long-term antiplatelet and anticoagulant therapy in patients with IHD is strongly associated with an increased risk of peptic ulcer disease and gastrointestinal hemorrhage, particularly in those receiving combined regimens. Prior ulcer history and NSAID use significantly elevate bleeding risk.

## Introduction

Ischemic heart disease (IHD) continues to be a major global health burden, contributing substantially to premature mortality and long-term disability [1]. The main pillar of treatment and secondary prevention in these patients is permanent use of anticoagulant and antiplatelet therapy, which minimizes the frequent thrombotic events by disrupting the platelet activation procedure and the coagulation cascade. The benefits of aspirin, clopidogrel, ticagrelor, warfarin, as well as more recent direct oral anticoagulant (DOAC) are all clear cardiovascular advantages [2]. However, the positive effect of these treatments should never be disregarded in comparison with the negative one, gastrointestinal (GI) bleeding, which is a serious clinical problem, especially in groups at risk, including peptic ulcer disease (PUD) [3]. A multifactorial etiology is associated with peptic ulcer disease, which is a condition characterized by mucosal damage of the stomach or duodenum, and it goes beyond the muscularis mucosae. Classical risk factors include *Helicobacter pylori* infection, use of nonsteroidal anti-inflammatory drugs (NSAIDs), smoking, and old age [4]. Chronic low-cardioprotective dose aspirin interferes with the production of gastric prostaglandins, resulting in the loss of mucosal protection, gastric acid damage, and ulceration [5]. The risk of developing clinically significant bleeding increases significantly when used together with other platelet inhibitors or anticoagulants [6]. Gastrointestinal bleeding with the background of PUD is a common complication, and at the same time, it is potentially fatal, particularly in patients with IHD [7].

Furthermore, the need for uninterrupted antithrombotic therapy in patients with recent coronary interventions or atrial fibrillation places clinicians in a therapeutic dilemma: stopping therapy heightens the risk of catastrophic cardiovascular events, while continuing therapy in the presence of ulceration increases the risk of severe hemorrhage [8]. The relationship between therapy duration, ulcer pathology, and patient characteristics must be precisely understood in order to strike a balance between these competing risks [9]. Epidemiological evidence indicates that the incidence of major GI bleeding among patients on dual antiplatelet therapy ranges from 1% to 3% annually, with higher rates in elderly individuals and those with prior ulcer disease. The risk significantly rises when anticoagulants are added to this regimen, frequently exceeding 5% per year [10]. The degree of platelet inhibition, the type of anticoagulant, renal and hepatic function, and the presence of underlying mucosal disease all influence this risk, which is not uniform. Despite advances in cardiovascular care, GI hemorrhage remains one of the most frequent causes of hospitalization among patients treated with long-term anti-thrombotics [11]. Despite extensive global research on antithrombotic-related GI complications, a clear gap remains in distinguishing bleeding risk from ulcer prevalence, as many studies focus primarily on hemorrhage while underreporting the burden of asymptomatic or early-stage PUD.

The main aim of the study is to determine the association between peptic ulcer disease and gastrointestinal hemorrhage with long-term use of antiplatelet and anticoagulant therapy in patients with ischemic heart disease, and to evaluate the magnitude of bleeding risk, contributing factors, and clinical outcomes in this population.

## Materials and methods

This was a cross-sectional analytical study conducted at a tertiary care hospital in Azad Kashmir, Pakistan, from June 2024 to May 2025. A total of 210 patients were included in the study.

The sample size was calculated using the WHO sample size calculator (https://cdn.who.int/media/docs/default-source/ncds/ncd-surveillance/steps/sample-size-calculator.xls), considering the anticipated prevalence of gastrointestinal bleeding in patients receiving long-term antithrombotic therapy, a confidence level of 95%, and a margin of error of 5%. This calculation yielded a required sample size of 210, which was enrolled consecutively. The sample size was calculated using the WHO formula for cross-sectional studies:

n=Z^2^×p×(1−p)​/d^2^

where Z=1.96, p=0.16, and d=0.05, yielding n=206. After adjusting for potential data loss, the final sample size was 210 participants.

Non-probability consecutive sampling was employed to recruit patients who fulfilled the eligibility criteria until the required sample size was achieved. Long-term therapy was defined as continuous use of antiplatelet and/or anticoagulant agents for at least six months. The six-month threshold was chosen based on established cardiology guidelines indicating that the majority of adverse GI effects from antithrombotic therapy occur after cumulative exposure over several months. Therapy duration was verified through hospital medical records, pharmacy prescription logs, and treatment cards, and was cross-checked with patient recall to ensure accuracy.

All patients aged 18 years and older with established ischemic heart disease were eligible for inclusion if they had been receiving long-term antiplatelet therapy (such as aspirin or clopidogrel) and/or anticoagulant therapy (such as warfarin or direct oral anticoagulants) for at least six months. Patients with pre-existing chronic liver disease, hematological bleeding disorders, or malignancies were excluded from the study. Additionally, those with incomplete or unreliable medical records were excluded.

After informed consent was obtained, data were collected using a predesigned structured proforma. Information was recorded on demographic characteristics (such as age and gender), comorbidities (including diabetes mellitus, hypertension, and chronic kidney disease), type and duration of antithrombotic therapy, and history of NSAID use or prior peptic ulcer disease. Patients presenting with clinical suspicion of gastrointestinal bleeding were evaluated with standard investigations, and upper gastrointestinal endoscopy was performed to confirm the presence of peptic ulcer disease and/or hemorrhage.

The primary outcome was the frequency of peptic ulcer disease and gastrointestinal hemorrhage among patients on long-term antiplatelet and anticoagulant therapy. Secondary outcomes included the severity of bleeding, classified as mild, moderate, or severe based on clinical presentation and endoscopic findings, and the association of type and duration of therapy with gastrointestinal complications.

All data were analyzed using SPSS 26 (IBM, Inc., Armonk, US). Quantitative variables such as age and duration of therapy were expressed as mean±standard deviation, while qualitative variables, including gender, presence of peptic ulcer disease, and occurrence of gastrointestinal hemorrhage, were presented as frequencies and percentages. Associations between categorical variables were determined using the Chi-square test, while an independent t-test was applied for continuous variables. A p-value of ≤0.05 was considered statistically significant.

## Results

A total of 210 patients with ischemic heart disease were included in the study. The mean age was 58.4±9.6 years. Males constituted the majority of the cohort (n=132; 62.9%), while females accounted for 37.1% (n=78). Hypertension was the most common comorbidity, affecting 118 patients (56.2%), followed by diabetes mellitus in 92 patients (43.8%) and chronic kidney disease in 28 patients (13.3%). A history of smoking was present in 67 individuals (31.9%). Regarding treatment, half of the patients (n=105; 50%) were on dual antiplatelet therapy, while 65 (31.0%) received single antiplatelet therapy, and 40 (19.0%) were taking a combination of antiplatelet and anticoagulant therapy (Table 1).

**Table 1:** Baseline demographic and clinical characteristics of the patients (n=210)

Variable	Category	n (%)	95% CI
Age (years), mean±SD		58.4±9.6	57.1-59.7
Gender	Male	132 (62.9%)	56.4-69.0
	Female	78 (37.1%)	31.0-43.6
Hypertension	Present	118 (56.2%)	49.5-62.7
Diabetes mellitus	Present	92 (43.8%)	37.3-50.5
Chronic kidney disease	Present	28 (13.3%)	9.1-18.7
Smoking	Present	67 (31.9%)	26.0-38.3
Type of therapy	Single antiplatelet	65 (31.0%)	25.3-37.4
	Dual antiplatelet	105 (50.0%)	43.3-56.7
	Antiplatelet + anticoagulant	40 (19.0%)	14.0-24.8

Peptic ulcer disease was diagnosed in 64 patients (30.5%). Its frequency was significantly higher among those on combined antiplatelet and anticoagulant therapy (45%, n=18) compared to dual antiplatelet therapy (32.4%, n=34) and single antiplatelet therapy (18.5%, n=12; p=0.003). Gastrointestinal hemorrhage was observed in 49 patients (23.3%), with the highest occurrence again in patients on combined therapy (45%, n=18), followed by dual antiplatelet therapy (21.9%, n=23) and single antiplatelet therapy (12.3%, n=8; p=0.001; see Table 2).

**Table 2:** Frequency of peptic ulcer disease and gastrointestinal hemorrhage (n=210) * Chi-square test was applied; p≤0.05 was considered statistically significant GI - gastrointestinal

Outcome	Single antiplatelet therapy (n=65)	Dual antiplatelet therapy (n=105)	Antiplatelet + anticoagulant therapy (n=40)	Total (n=210)	p-value
Peptic ulcer disease, n (%)	12 (18.5%)	34 (32.4%)	18 (45.0%)	64 (30.5%)	0.003*
GI hemorrhage, n (%)	8 (12.3%)	23 (21.9%)	18 (45.0%)	49 (23.3%)	0.001*

Multivariate logistic regression analysis identified several independent predictors of GI hemorrhage. A prior history of peptic ulcer disease (OR 2.6, 95% CI: 1.3-5.1, p=0.004) and NSAID use (OR 3.1, 95% CI: 1.5-6.4, p=0.002) were strongly associated with bleeding. Type of therapy was also significant: dual antiplatelet therapy was associated with twice the odds of bleeding (OR 2.0, 95% CI: 1.1-3.8, p=0.02), while the combination of antiplatelet and anticoagulant therapy carried the highest risk (OR 4.2, 95% CI: 2.1-8.4, p<0.001). Other factors, such as age above 65 years, male gender, hypertension, and diabetes mellitus, were not statistically significant (Table 3).

**Table 3:** Logistic regression analysis for predictors of gastrointestinal hemorrhage * p-value ≤0.05 was considered statistically significant NSAID - nonsteroidal anti-inflammatory drug

Variable	Odds ratio (OR)	95% confidence Interval	p-value
Age >65 years	1.4	0.7-2.8	0.28
Male gender	1.1	0.6-2.0	0.71
Hypertension	1.3	0.7-2.4	0.38
Diabetes mellitus	1.6	0.9-3.1	0.09
Prior ulcer history	2.6	1.3-5.1	0.004*
NSAID use	3.1	1.5-6.4	0.002*
Dual antiplatelet therapy	2.0	1.1-3.8	0.02*
Antiplatelet + anticoagulant therapy	4.2	2.1-8.4	<0.001*

Out of the 49 patients who experienced hemorrhage, 28 (57.1%) had mild bleeding, 15 (30.6%) had moderate bleeding, and six (12.3%) suffered severe hemorrhage. When stratified by therapy groups, mild hemorrhage was most common across all groups, while severe hemorrhage was relatively rare, with no statistically significant difference in severity distribution among single, dual, or combined therapy groups (p>0.05; see Table 4).

**Table 4:** Severity of gastrointestinal hemorrhage across therapy groups (n=49) Chi-square test was applied; no statistically significant difference was found across groups.

Severity of hemorrhage	Single antiplatelet therapy (n=8)	Dual antiplatelet therapy (n=23)	Antiplatelet + anticoagulant therapy (n=18)	Total (n=49)	p-value
Mild, n (%)	5 (62.5%)	14 (60.9%)	9 (50.0%)	28 (57.1%)	0.62
Moderate, n (%)	2 (25.0%)	7 (30.4%)	6 (33.3%)	15 (30.6%)	0.81
Severe, n (%)	1 (12.5%)	2 (8.7%)	3 (16.7%)	6 (12.3%)	0.55

## Discussion

This study investigated the relationship between long-term use of antiplatelet and anticoagulant therapy and the occurrence of peptic ulcer disease (PUD) and gastrointestinal (GI) hemorrhage in patients with ischemic heart disease (IHD). Among the 210 patients enrolled, nearly one-third were found to have peptic ulcer disease, while almost one-fourth developed gastrointestinal bleeding. The risk was highest among those receiving combined antiplatelet and anticoagulant therapy, indicating a strong additive effect of these drugs on gastrointestinal mucosal vulnerability. Our findings are in line with those of previous studies that have shown that patients receiving dual or triple antithrombotic therapy have a higher risk of upper GI bleeding. Lanas and Chan noted that even low-dose aspirin significantly increases the risk of peptic ulcer bleeding, and the addition of clopidogrel or oral anticoagulants multiplies this risk [12]. The 45% prevalence of hemorrhage in patients receiving combined therapy in our cohort aligns with global evidence showing higher bleeding rates when anticoagulants are added to dual antiplatelet therapy. Risk factor analysis in our study further demonstrated that a prior history of peptic ulcer disease was a significant predictor of GI hemorrhage. This observation underscores the importance of comprehensive risk stratification before initiating long-term antithrombotic therapy. Previous research has identified these factors, along with advanced age and comorbid conditions, as key contributors to bleeding risk [13]. 

According to large cohort analyses, NSAIDs synergistically enhanced mucosal injury in patients who were already vulnerable due to platelet inhibition. Our study's independent association with hemorrhage echoes these findings. Interestingly, while older age and diabetes were more frequent in patients with bleeding complications, they did not reach statistical significance as independent predictors in our regression model [14]. This could be attributed to sample size limitations or the predominant effect of more direct risk factors such as prior ulcer history and drug combinations. However, the clinical importance of age-related frailty and comorbidity burden should not be underestimated, as other multicenter trials have demonstrated that age >65 years is a consistent bleeding risk enhancer [15]. According to our study's severity distribution, the majority of bleeding events were mild to moderate, with 12.3% of cases requiring transfusions and endoscopic procedures. Severe hemorrhage in IHD patients carries additional risk because it can precipitate myocardial ischemia, arrhythmias, and hemodynamic compromise. This dual burden of cardiovascular instability and hemorrhagic shock has been highlighted in prior registries, where mortality from GI bleeding was disproportionately high among cardiac patients compared to the general population [16,17].

This study's findings have several clinical implications. First, they support guidelines recommending gastroprotective measures for high-risk patients on long-term antithrombotic therapy. Proton pump inhibitors (PPIs) have been shown to significantly reduce upper GI bleeding without compromising cardiovascular protection. Their use should be seriously considered, especially in patients who have had ulcer disease in the past, are taking NSAIDs at the same time, or need to take both medications at the same time. Second, the study highlights the importance of avoiding unnecessary NSAID prescriptions in IHD patients already on platelet inhibitors, as this combination was strongly associated with bleeding events. Finally, risk stratification tools should be routinely applied before initiation of therapy, allowing clinicians to balance the benefits of thromboprophylaxis against bleeding hazards.

Despite its strengths, this study is not without limitations. Because it is cross-sectional, it cannot establish causality, and there may be residual confounding from unmeasured variables such as adherence, genetic predisposition, and dietary factors. Additionally, the study was conducted in a single center, which may limit generalizability. Endoscopic evaluation was performed only in symptomatic patients, so subclinical ulcers might have been underestimated. Future studies with prospective, multicenter designs and larger sample sizes are warranted to validate these findings and to develop context-specific guidelines for gastroprotection in high-risk IHD patients.

## Conclusions

It is concluded that long-term antiplatelet and anticoagulant therapy in patients with ischemic heart disease is significantly associated with increased rates of peptic ulcer disease and gastrointestinal hemorrhage, with the highest risk observed among those receiving combined antiplatelet-anticoagulant regimens. Prior ulcer history and concurrent NSAID use were strong independent predictors of bleeding, emphasizing the importance of individualized risk assessment before initiating or intensifying therapy. However, because this study employed a cross-sectional design, causal relationships cannot be established, and the findings should be interpreted with appropriate caution. The results support the use of routine gastroprotection such as PPIs in high-risk individuals and align with current international guidelines that recommend minimizing unnecessary NSAID exposure and carefully evaluating the need for combination therapy.
